# Nontraditional Roles of DNA Polymerase Eta Support Genome Duplication and Stability

**DOI:** 10.3390/genes14010175

**Published:** 2023-01-09

**Authors:** Kristin A. Eckert

**Affiliations:** Gittlen Cancer Research Laboratories, Department of Pathology, Penn State University College of Medicine, 500 University Drive, Hershey, PA 17036, USA; kae4@psu.edu

**Keywords:** POLH, XP-V, common fragile site, replication stress, difficult-to-replicate sequence, somatic hypermutation, D-loops, senescence, telomeres

## Abstract

DNA polymerase eta (Pol η) is a Y-family polymerase and the product of the *POLH* gene. Autosomal recessive inheritance of *POLH* mutations is the cause of the xeroderma pigmentosum variant, a cancer predisposition syndrome. This review summarizes mounting evidence for expanded Pol η cellular functions in addition to DNA lesion bypass that are critical for maintaining genome stability. In vitro, Pol η displays efficient DNA synthesis through difficult-to-replicate sequences, catalyzes D-loop extensions, and utilizes RNA–DNA hybrid templates. Human Pol η is constitutively present at the replication fork. In response to replication stress, Pol η is upregulated at the transcriptional and protein levels, and post-translational modifications regulate its localization to chromatin. Numerous studies show that Pol η is required for efficient common fragile site replication and stability. Additionally, Pol η can be recruited to stalled replication forks through protein–protein interactions, suggesting a broader role in replication fork recovery. During somatic hypermutations, Pol η is recruited by mismatch repair proteins and is essential for V_H_ gene A:T basepair mutagenesis. Within the global context of repeat-dense genomes, the recruitment of Pol η to perform specialized functions during replication could promote genome stability by interrupting pure repeat arrays with base substitutions. Alternatively, not engaging Pol η in genome duplication is costly, as the absence of Pol η leads to incomplete replication and increased chromosomal instability.

## 1. Introduction

Human DNA polymerase eta (Pol η) was identified in 1999 as the product of the *POLH* gene through elegant biochemical analyses [1]. Masutani et al. demonstrated that cell lines from xeroderma pigmentosum variant (XP-V) patients had mutations in *POLH* and that a recombinant human Pol η protein corrected the UV-lesion translesion synthesis (TLS) defect present in XP-V cell extracts. Since this discovery, the majority of the research regarding Pol η’s cellular functions has focused on DNA damage response, particularly after UV irradiation. However, research during the past 10 years has provided mounting evidence for Pol η’s expanded roles in genome duplication, outside of lesion bypass. Here, I review discoveries of the human Pol η’s non-TLS biochemical activities, distinct damage-independent Pol η regulation pathways, and Pol η’s functions in DNA replication. Together, this evidence suggests that Pol η activity is continually required in mammalian cells to ensure complete genome duplication and maintain genome stability. Accordingly, I will refer to Pol η as a “specialized polymerase” rather than a TLS polymerase to acknowledge the importance of these varied cellular functions. The review focuses almost exclusively on studies performed using human and mouse model systems.

## 2. In Vitro Characterization of Pol η Properties

### 2.1. Biochemical Activities Outside of TLS

Pol η is a Y-family polymerase of the RAD30A branch and is highly conserved in eukaryotes [2]. The N-terminal half of the protein encodes the catalytic (DNA synthesis) domain. For a recent review of Pol η’s structure and associated functions, see [3]. Impediments to genome replication arise from both endogenous and exogenous sources. Replicative polymerases encounter many difficult-to-replicate sequences (DiToRSs), which are regions of the genome that hinder DNA synthesis elongation (reviewed in [4]). Endogenous replication fork barriers include naturally arising physical obstacles, such as protein–DNA complexes or transcription–replication collisions (e.g., R-loops), and non-B DNA secondary structures formed within repetitive regions, common fragile sites (CFSs), and telomeres. Below, the biochemical activities of Pol η that are important for DiToRS replication are described.

#### 2.1.1. DiToRSs within Common Fragile Sites

The CFS FRA16D contains AT-rich “flexibility” regions that are associated with replication fork pausing and chromosomal breakage (reviewed in [5]). Our laboratory demonstrated that the [AT/TA]_34_ microsatellite within the Flex 1 region impairs in vitro DNA synthesis by the replicative human Pol δ holoenzyme [6]. We also identified additional DiToRSs within FRA16D that are inhibitory to human replicative DNA polymerases, including a long mononucleotide [A/T]_28_ repeat (within the Flex 5 region) and quasi-palindrome (hairpin) structures formed by imperfect repeats [7,8]. Such DiToRSs inhibit in vitro DNA synthesis by the replicative polymerases α-primase and δ and inhibit DNA synthesis in cell-free human extracts [7].

Using the same DiToRS templates, our laboratory discovered that human Pol η is more efficient than Pol δ for the synthesis of both AT-rich repeats and quasipalindromes [9]. Furthermore, we designed a model of lagging strand replication with RFC-loaded PCNA that allows for high activity of both the human Pol δ holoenzyme and Pol η. Using this dual polymerase assay, we demonstrated that Pol η can exchange with Pol δ stalled at a DiToRS, thereby supporting complete CFS synthesis [10]. We also used this model to examine the impact of aphidicolin (Aph), an inhibitor of B-family polymerases [11]. The Pol δ holoenzyme synthesis was significantly inhibited by Aph in a dose-dependent manner, preventing replication past the DiToRS. Importantly, we showed that Pol η is aphidicolin-resistant and can rescue this stalled Pol δ synthesis to complete CFS replication [10]. It is worth noting that improved DiToRS synthesis in the dual polymerase assays was also measured in the presence of Pol κ, a related Y-family polymerase. The degree to which Pols η and κ perform overlapping versus distinct DiToRS synthesis functions is unknown.

#### 2.1.2. D-Loop Extension

During recombination-associated DNA synthesis, DNA polymerases must extend the 3′OH of an invading single DNA strand and continuously displace the downstream duplex DNA of the “D-loop” substrate to continue strand migration. McIlwraith et al. [12] used HeLa cell extract fractionation to examine the polymerases required for the extension of artificial D-loop substrates as a model for DNA synthesis that must proceed after an invading strand has initiated recombination. These authors showed that only Pols δ and η were required for D-loop extension and that this activity was defective in XP-V cell extracts. Further studies demonstrated that purified Pol η was able to capture the invading strand and promote D-loop primer extension [13]. In vitro, Pol η performs efficient D-loop primer extension synthesis in the absence of PCNA, but the presence of PCNA/RFC increases product length by up to 2000 nucleotides [14,15]. Pol η also performs D-loop extension efficiently when the invading strand encodes telomeric repeat sequences [16].

#### 2.1.3. Utilization of RNA-Containing Substrates

RNA is utilized for at least two functions during genome duplication: priming DNA synthesis and double-strand break (DSB) repair. RNA priming of the DNA occurs at each replication origin, at each lagging strand Okazaki fragment during replication elongation, and during the restart of stalled forks. These pathways require DNA polymerases to extend an RNA-primed DNA template (RNA/DNA substrate). RNA can also be used as a template for DSB repair (reviewed in [17]). This pathway requires the reverse transcriptase activity of the polymerases to synthesize DNA using an RNA template (DNA/RNA substrate). In addition, R-loops are three-stranded structures containing an RNA–DNA hybrid and a displaced DNA strand and can be found at centromeric and telomeric repeats and RNA polymerase pause sites (reviewed in [18]). In vitro, Pol η has biochemical activities that are consistent with roles in processing RNA–DNA hybrids. The Pol η’s catalytic fragment binds to DNA/DNA, RNA/DNA, and DNA/RNA primer–template substrates with similar affinities (K_d_~60 nM) [19]. During the extension of an RNA-primed DNA substrate, Pol η prefers the insertion of dNTP substrates over rNTP substrates, even when rNTPs are present at physiologically higher concentrations than dNTPs. Pol η is also a very efficient reverse transcriptase. Su et al. showed that Pol η fully elongates a DNA-primed RNA template, while maintaining its high selectivity (1000-fold) for dNTP incorporation over rNTP incorporation [19].

### 2.2. Accuracy of DNA Synthesis

Human cells encode 15 distinct nuclear DNA polymerases, and DNA polymerase fidelity (i.e., the accuracy displayed during DNA synthesis) is another biochemical property that is widely used to characterize different enzymes [20]. Replicative, B-family polymerases (Pol δ and Pol ε) have high accuracy due, in part, to the presence of a very active 3′ to 5′ proofreading exonuclease, while proofreading-deficient enzymes, such as the specialized Y-family polymerases, generally display lower accuracy. For example, our laboratory developed the in vitro herpes simplex virus *thymidine kinase* (HSV-*tk*) polymerase fidelity assay to directly measure and compare polymerase errors created during the synthesis of various DNA sequences [21]. During the synthesis of the “housekeeping” HSV-*tk* gene sequences, the human Pol η is ~80-fold less accurate for base substitution errors and 140-fold less accurate for indel (insertion/deletion) errors than the human Pol δ4 replicative enzyme [22]. Among base substitutions, Pol η creates errors at the T and A bases at a higher frequency than errors at the G or C bases [22]. These results are consistent with other studies and the general characterization of Pol η as a low-fidelity polymerase [23,24].

#### 2.2.1. Microsatellite Stability

Microsatellites are tandemly repeated DNA sequences located throughout the human genome. When present in pure repeated arrays above a threshold length, the microsatellites display a characteristically high mutation frequency and a high degree of genetic variation among individuals [25,26]. Interruption mutations that disrupt perfect repeats decrease the frequency of microsatellite mutations genome-wide [27]. Using our in vitro HSV-*tk* polymerase fidelity assay, we demonstrated that such beneficial interruption errors are created most often by Y-family polymerases but rarely by replicative polymerases [27]. For example, during the synthesis of [GT]_n_ and [TC]_n_ microsatellites, Pol η produced a characteristically high proportion of base substitution errors within the repeated microsatellites. These interrupting base substitutions are created very efficiently by Pol η, and the enzyme often creates multiple errors within a single DNA synthetic event. In addition, we showed a novel signature of Pol η errors produced during the synthesis of A_3_TA_4_/T_3_AT_4_ interrupted microsatellite templates, in which additional Pol η errors create a DNA synthesis product that is more random in sequence than the starting template sequence [27].

#### 2.2.2. G-Quadruplex Synthesis

G-quadruplexes (G4s) are a specific type of non-B DNA structure formed through Hoogsteen base pairing of repeated guanines within tracts separated by loop sequences (reviewed in [28]). In vitro, the catalytic cores of human Pol η and Pol κ efficiently bind G4-containing DNA substrates [29]. Using our in vitro HSV-*tk* polymerase fidelity assay, we observed unique human polymerase error signatures during the synthesis of G4 motif templates [30]. Polymerase errors occurred within, immediately flanking, and encompassing the G4 motifs; however, the distribution and types of errors were dependent on both the polymerase and the G4 motif sequence and/or topology. In silico analyses of Pol η base substitutions and indel errors within a thermally unstable G4 motif that has an antiparallel strand topology showed that the resulting mutant sequences generally maintained G4 formation and stability. In contrast, Pol η frequently deleted large portions of a thermally stable G4 motif that has a parallel strand topology, often involving the surrounding sequence and eliminating the G4-forming potential of the resulting mutant sequences. Interestingly, Pol η also created a significant number of errors, specifically within the 3′ sequences, flanking a thermostable, parallel G4 motif.

## 3. Constitutive and Replication Stress-Induced Pol η Regulation

The C-terminal half of the Pol η protein is regulatory in nature. The final 120 amino acids of the human Pol η protein encode the nuclear localization sequence and are necessary for Pol η foci formation after UV treatment [31]. The C-terminal region contains several additional protein–protein interaction and post-translational regulatory domains [3,20]. For an in-depth review of DNA polymerase’s constitutive and damage-induced regulation, including POLH/Pol η, see [20].

### 3.1. Constitutive Regulation in Unperturbed Cells

The *POLH* gene is positively regulated by the p53 transcription factor in human cells, and the overexpression of TP53 increases Pol η’s mRNA levels [32]. Higher *POLH* mRNA and Pol η protein levels are observed in TP53 proficient cells compared to deficient cells [33]. Analyses of mRNA expression in U2OS cells showed a mild increase in *POLH* mRNA from G1 through S to G2 [34]. The Pol η protein has a short half-life (<30 min in RKO cells) and is degraded by a proteosome [35]. Protein stability is regulated in a polyubiquitin-independent manner by the E3 ligase Pirh2 [35] and in a polyubiquitin-dependent manner by Mdm2 [36].

Post-translational modifications are important Pol η regulators. Pol η has multiple phosphorylation sites, and the overall phosphorylation status of Pol η changes in a cell cycle-dependent manner, increasing at the end of the S phase and reaching its maximum at the metaphase–anaphase transition [37]. CDK2 mediates the phosphorylation of Ser 687, which is maximal at the G2/M transition, and this phosphorylation regulates the Pol η’s protein levels [37].

Using the iPOND technique, Despras et al. showed that Pol η travels with replication forks in unperturbed human cells [38]. This fork association requires K163 SUMOylation by the E3 ligase PIAS1, and SUMOylation is dependent on a direct interaction between Pol η and Rad18 [38]. Mutations in the Pol η’s modification site (K163R) decrease spontaneous foci formations in S-phase cells and abolishes the recruitment of Pol η to replication forks in unperturbed human cells [38]. Pol η contains a C-terminal Ub-binding (UBZ) domain, and also is monubiquitylated in a region directly adjacent to the UBZ domain [39,40]. Endogenous Ub–Pol η is detected in unperturbed human cells [40]. This ubiquitylation has been mapped to lysines within a region containing the Pol η nuclear localization sequence and a broad PCNA-interacting surface. An intramolecular interaction between the C-terminal Ub moiety and the UBZ domain has been proposed to block Pol η’s interaction with other Ub proteins [40]. For instance, the interaction between Pol η and Ub-PCNA occurs through the Pol η UBZ domain. One hypothesized function of monoUb–Pol η is to modulate its residence time in replication foci. More recently, an interaction of Pol η with Ub-H2A was shown to be important for the recruitment of Pol η to chromatin in unperturbed cells [41], demonstrating that Pol η interactions with Ub-containing proteins other than Ub-PCNA can regulate the localization of Pol η.

### 3.2. Regulation in Response to Replication Stress

In human cell studies, two drugs that do not directly form DNA adducts, Aph and hydroxyurea (HU), are routinely used to experimentally induce replication stress. As mentioned above, Aph is a specific inhibitor of B-family DNA polymerases (Pols α, δ, ε) but not of the Y-family Pols η and κ [10]. HU is a ribonucleotide reductase inhibitor that causes depletion of dNTP pools [42,43].

Endogenous Pol η displays significant mRNA and protein upregulation in response to experimentally induced replication stress [33]. The *POLH* transcriptional response to Aph is p53-independent. Under replication stress, Pol η is relocalized to chromatin and recruited to nuclear foci containing PCNA [33]. The replication stress induced by HU does not alter the cellular levels of monoUb–Pol η, which contrasts with DNA-damaging agents, such as UV, which reduce the levels of monoUb–Pol η [40]. Nonetheless, the UBZ domain is required for Pol η to access chromatin in response to replication stress [33]. The Pol η SUMOylation is increased after the induction of replication stress, and the expression of the K163R mutant (which abrogates SUMOylation) cannot rescue the Aph-sensitive phenotypes of XPV cells [38].

The ATR pathway is required to ensure the completion of DNA replication and genome stability under replication stress conditions. ATR mediates the Pol η Ser 601 phosphorylation when Pol η is localized to chromatin, and this phosphorylation requires the Pol η UBZ domain and interaction with Rad18 [44]. In MRC5 cells, overexpression of a *POLH* (S601E, S587E, and T617E) mutant that mimics ATR and CDK2 phosphorylation leads to the formation of more Pol η foci than wild-type cells in the absence of DNA damage, which is consistent with enhanced retention of the mutant on chromatin [45]. The purified mutant protein forms a highly stable complex with Ub-PCNA.

## 4. Pol η Functions in DNA Replication

In the absence of exogenous DNA damage, genome-wide DNA replication studies in S. cerevisiae have shown that Pol η can compete with Pols *δ* and *α* and contribute to lagging strand DNA synthesis [46]. Numerous studies in human cells have revealed that specialized polymerases are required to complete genome duplication under various conditions. This research examining Pol η is summarized below, relating the cellular processes to the non-TLS biochemical activities of the enzyme (Section 2) and to Pol η regulation (Section 3).

### 4.1. Common Fragile Sites (CFSs)

Current evidence shows that Pol η is required to maintain genome stability and prevent under-replicated DNA at CFS loci. Pol η-deficient human cells display increased formation of spontaneous chromosomal abnormalities and CFS breakage, which is enhanced under replication stress conditions [47]. Recently, Twayana et al. demonstrated directly that Pol η-deficient cells increase replication fork pausing (relative to Pol η-proficient cells) at several CFS regions, both within and outside AT-rich fragility cores [48]. Mechanistically, Pol η is enriched at CFS loci after replication stress [9]. FANCD2 is a replication protein that facilitates CFS replication [49], and the Pol η localization to chromatin in response to HU is mediated by FANCD2 [50].

Under replication stress conditions, Pol η-deficient cells display RPA foci, 53BP1 nuclear bodies, and an increased number of EdU+ mitotic cells, which is consistent with incomplete genome duplication [9]. Pol η-deficient cells in the presence of replication stress also display defective G2/M phase progression and increased activation of the ATR/Chk1 axis [9,33]. This cell cycle response differs from the UV damage response, wherein Pol η-deficient cells stall in the S phase. The presence of Pol η continues to be critical during recovery from replication stress as Pol η-deficient cells display aberrant phenotypes even after Aph release, including delayed cell cycle progression, increased apoptosis and EdU+ mitotic cells, and decreased cell survival [33].

Elegant single molecule studies using purified S. cerevisiae replication proteins have revealed that the eukaryotic replisome is a highly dynamic structure [51]. Based on their study, Lewis et al. proposed that a concentration-dependent polymerase exchange within the replisome ensures continuous fork progression under stress conditions and is an important mechanism for genome stability. Taken together with the studies described above, the available evidence supports the following model for Pol η’s critical involvement in CFS replication. Pol η can contribute to lagging strand synthesis and is present at the replication fork under unperturbed conditions. In response to replication stress, Pol η protein levels are increased, and Pol η is post-translationally modified. Consequently, Pol η is recruited to CFS loci and nuclear foci containing PCNA, possibly as a component of the replisome. Biochemically, Pol η extends replication intermediates that are stalled or abandoned by Pol *δ*, for instance at DiToRSs, and carries out Aph-resistant DNA synthesis. In this way, Pol η generates the optimal DNA primer–templates for replicative polymerases to resume replication fork elongation. Because replicative DNA polymerase synthesis is slow within CFSs and other DiToRSs even in the absence of replication stress [52], Pol η might have an ongoing role in late S/G2 pathways to complete genome duplication.

### 4.2. Stalled Replication Forks

Pol η may potentially participate more broadly in replication through the restart of stalled replication forks. For instance, replication stress induced by the c-Myc oncogene overexpression in U2OS cells causes Pol η relocalization to replication foci [53]. Consistent with its role in replication fork rescue, POLH knockdown in this system enhanced c-Myc-induced replication stress, measured in DNA fiber assays as decreased fork velocity and increased fork asymmetry. Replication stress induced in human cells by HU or Aph causes Pol η relocalization into foci with PALB2, RAD51, and BRCA2 [54]. Mutations of PALB2 or BRCA2 decreased HU-induced Pol η foci formation. Because this co-localization was not observed after ionizing radiation, Pol η is likely being recruited to stalled replication forks rather than DSBs [54]. Pol η also may assist in resolving replication forks stalled by transcription–replication fork collisions. In a model using double-minute (DM) chromosomes from the c-Myc locus, which are small circular fragments of extrachromosomal DNA, Watanabe et al. discovered that replication forks pause within a DNA region, which is characterized by high transcription and the formation of lncRNAs [55]. Extensive analyses showed that R-loops are formed within the stalled regions and that RPA, BRCA2, and Pol η are all recruited to the same region. Importantly, knockdown of POLH resulted in a 40% reduction in DM formation, which is consistent with a requirement of Pol η in replicating this region. These two studies are consistent with a model in which PALB2 and BRCA2 target Pol η to stalled replication forks. One possibility, based on the biochemical studies described above, is that PABLB2 and BRCA2 target Pol η to the 3′ end of an invading DNA strand during fork restart, after which Pol η participates with Pol *δ* in efficient D-loop extension syntheses.

Clues to a potential alternative mechanism for Pol η resolution of stalled forks can be found in studies of S. cerevisiae. RNaseH1 and RNaseH2 are riboendonucleases that cleave RNA in RNA–DNA hybrids, initiating the ribonucleotide excision repair pathway [18]. Yeast RNase H1/2-deficient cells are hypersensitive to HU treatments. Interestingly, Meroni et al. showed that almost all of the HU toxicity in RNase H1/2-deficient yeast is dependent on Pol η [56]. This result suggests that Pol η facilitates replication of RNA-containing DNA and/or that Pol η actively incorporates ribonucleotides under HU conditions, both of which become lethal when all RNaseH activity is absent. Whether this toxicity is related to Pol η’s ability to utilize RNA-containing primers or RNA templates has not been tested. Importantly, this yeast study provides an alternative pathway to the recombination/D-loop extension model for understanding Pol η’s role in completing genome duplication under replication stress conditions.

### 4.3. Telomere Replication

Telomeres are inherently difficult to replicate due to their highly repetitive sequence composition and propensity to form structures such as G-quadruplexes, RNA–DNA hybrids, and D-loops. The alternative lengthening of telomeres (ALT) pathway is a mechanism of telomere maintenance in telomerase-deficient cells that involves chromatin remodeling and interchromosomal recombination within telomeric repeats (see [57] for a recent review). Pol η was identified as one of 139 proteins that make up the telomere proteome in ALT-positive (ALT+) U2OS cells [16]. Pol η localization to telomeres is partially RAD18-, PALB2-, and TRF1-dependent and abolished by Pol η C-terminal domain mutations [16]. Pol η knockdown in ALT+ cells increases sister chromatid exchange at the telomeres and the appearance of fragile telomeres, especially after an Aph treatment. While this study indicates that Pol η directly functions in the ALT pathway to maintain telomere integrity, the precise mechanisms are not fully understood. In ALT+ cells, the lncRNA TERRA hybridizes to the C-rich strand of telomeric repeats, forming R-loops in a RAD51-dependent manner [18]. Therefore, several biochemical activities of Pol η may play a role in ALT+ telomere maintenance, including DiToRS synthesis, D-loop extension, and utilization of RNA–DNA hybrids.

## 5. Cell Fate

Pol η levels may be important for cell fate regulation. A two-year study of *Polh*^−/−^ mice found no increased spontaneous tumorigenesis but, instead, revealed an increased level of senescence in the adipose tissues of *Polh*^−/−^ mice as early as 4 weeks of age [58]. Intriguingly, *Polh*^−/−^ adipocytes displayed increased DNA damage and increased levels of the pro-inflammatory cytokines IL6 and TNFα. *Polh* knockdown in mouse fibroblasts was sufficient to increase p16 levels and increase senescence-associated β-galactoside activity [58]. *POLH* depletion in hTERT-BJ5a human fibroblasts is also sufficient to increase p16 levels and induce a senescence-like growth arrest [59]. The mechanisms by which Pol η deficiency leads to senescence are unknown.

## 6. Generating Diversity: Somatic Hypermutation

Somatic hypermutation (SHM) of immunoglobulin variable (V_H_) genes is necessary to produce a full repertoire of B-cell antigen binding sites during the adaptive immune response (see [60] for a recent review of the mechanisms). SHM usurps the base excision and mismatch repair pathways to target a high frequency of base substitution mutations within a specific genome region. DNA synthesis by low-fidelity polymerases is needed to create a high frequency of both transition and transversion mutations at the A:T and G:C basepairs. The role of Pol η in SHM was first reported by Zeng et al. [61], who measured V_H_ gene mutations from peripheral blood lymphocytes of XP-V and control patients. While the frequency and distribution of the mutations were not altered, the authors measured a statistically significant decrease in the proportion of base substitutions at the A:T basepairs. A more recent study of two XP-V patient cohorts confirmed the significant decrease in Ig V_H_ gene A:T basepair mutations and showed that the percentage of A:T basepair mutations created in these patients is directly correlated with the *POLH* gene mutation and corresponding level of Pol η protein expression [62]. An in-depth analysis of Pol η errors created in vitro is consistent with the error specificity and hotspot DNA sequence contexts observed during in vivo SHM at Ig V_H_ genes [24]. In *Polh*^−/−^ mice, SHM-generated A:T basepair mutations are also severely reduced, especially the A to G and T to C transitions [63]. The mechanism for targeting Pol η to V_H_ genes during SHM is not fully understood. Mismatch repair proteins are involved in one arm of SHM, and Pol η directly interacts with MLH1 through its N-terminal region, forming a complex with MutLα and MutSα in HeLa cells [64]. Consistent with Pol η recruitment through MMR, A:T mutations are completely lacking in *Msh2*^−/−^*Polh*^−/−^ mice [65]. Based on the available evidence, Pol η is considered to be the sole polymerase contributing to A:T basepair mutations during SHM [65].

## 7. Conclusions and Perspective

Research over the past 10 years has provided clear evidence that Pol η plays critical roles in genome duplication outside of TLSs, particularly under replication stress conditions. The cellular requirement for Pol η to complete CFS replication is well supported by in vitro and ex vivo evidence. The extent to which Pol η participates in the replication of other genome regions enriched in DiToRSs, such as centromeres and telomeres, remains to be elucidated. Moreover, the extent of Pol η’s biochemical activities that are required for genome stability has not been fully characterized. In particular, the ability of Pol η to perform D-loop extension hints at a larger role in the resolution of stalled forks, such as recombination-mediated fork restart. Similarly, whether cellular functions rely on Pol η’s ability to extend RNA-primed DNA templates or DNA-primed RNA templates (reverse transcriptase activity) is not known. Perhaps, Pol η could replace Pol α/δ on the lagging strand and extend Okazaki fragments under specific cellular conditions (e.g., replication stress) or at specific genomic locations (e.g., repetitive sequences or CFSs). Because Pol η has low fidelity on undamaged DNA templates, an assumption in the field has been that cellular regulation must limit Pol η’s activity during replication to avoid mutagenesis. Contrary to this view, within the global context of the highly repetitive human genome, the recruitment of Pol η to perform specialized functions within difficult-to-replicate genome regions could, in the long term, promote genome stability by interrupting pure repeat arrays with base substitutions. Alternatively, not engaging Pol η in genome duplication could be costly in the long term, since the absence of Pol η leads to incomplete, under-replicated DNA entering mitosis and an increase in chromosomal instability.

A full understanding of the mechanisms regulating Pol η is critical for advancing our knowledge of human genome stability. Post-translational modifications and protein–protein interactions are important regulators of Pol η localization, and the manner of this regulation in response to replication stress differs from damage-induced Pol η regulation. Evidence for cellular targeting of Pol η to a specific genome location is found during somatic hypermutation, wherein Pol η is likely recruited by mismatch repair proteins for error-prone DNA synthesis at V_H_ gene regions. Additional evidence suggests that Pol η localization in response to replication stress is regulated by phosphorylation, SUMOylation, and direct interactions with several proteins, including PALB2, BRCA2, and FANCD2. Caution should be used, however, when inferring Pol η’s functions from evidence generated using a single human cell line. Pol η is regulated by the p53 pathway at both the transcriptional and post-translational levels, and the p53 pathway is widely mutated in tumor-derived cell lines or inactivated in SV40 or HPV-infected cell lines. Similarly, HeLa cells express a 55-kDa truncated form of Pol η that is missing the C-terminal regulatory domains [1], while U2OS cells are ALT+ and rely on Pol η for continued telomere maintenance and viability [16].

Pol η’s role in neoplastic development is likely complex, as this protein possesses functions that are both tumor suppressive (anti-tumorigenic) and oncogenic (pro-tumorigenic). The tumor suppressive functions in which Pol η deficiency promotes carcinogenesis are well understood. An accurate DNA lesion bypass mediated by Pol η prevents cancer development, as evidenced by the increased incidence of damage-induced skin tumors in XP-V patients [66] and Pol η-deficient mice [67]. Pol η also functions to prevent chromosomal instability at common fragile sites, as XP-V cells display increased CFS breakage and genome instability. Pol η is needed to create a full B-cell antibody repertoire through its essential role in A:T basepair somatic hypermutations, as both XP-V patients and *Polh*^−/−^ mice display reduced V_H_ gene diversity. However, whether the loss of Pol η leads to an impaired tumor cell immune response is unknown. On the other hand, emerging evidence of Pol η’s pro-survival cellular functions, particularly under various causes of replication stress, suggests that this polymerase may promote oncogenesis. Indeed, the *POLH* locus is primarily amplified in cancers, and this amplification is correlated with increased mRNA expression in esophageal, gastric, liver, ovarian, and melanoma tumors [4]. Increased *POLH* expression in tumors, relative to normal, has also been reported for kidney, colon, liver, breast, thyroid, gastric, and endometrial cancers [48]. Importantly, the mechanisms by which the presence of Pol η prevents cell senescence and promotes cell survival under replication stress conditions are yet to be discovered.
